# Investigation of Mechanical and Shrinkage Performance for Large-Size Cement-Stabilized Aggregates

**DOI:** 10.3390/ma17051027

**Published:** 2024-02-23

**Authors:** Chengwei Zhao, Tuo Huang, Xinglong Gao, Yahui Li, Li Lu

**Affiliations:** 1 Guangxi Transportation Science Engineering Construction Co., Ltd., Nanning 530007, China; gxjkgczxgs@163.com; 2 School of Traffic and Transportation Engineering, Changsha University of Science and Technology, Changsha 410114, China; gxl@stu.csust.edu.cn (X.G.); 22201060206@stu.csust.edu.cn (Y.L.); 202201140623@stu.csust.edu.cn (L.L.)

**Keywords:** large-size cement-stabilized macadam (LSCSM), dry shrinkage, temperature shrinkage, mechanical properties

## Abstract

Cement-stabilized macadam materials are widely utilized as semi-rigid base materials in road construction. However, conventional cement-stabilized macadam (CCSM) bases often develop shrinkage cracks during early construction and maintenance due to variations in humidity and temperature. Shrinkage cracks can subsequently result in reflective cracks in the asphalt pavement, significantly reducing the overall service life of the road. This study systematically evaluates the shrinkage and mechanical properties of large-size cement-stabilized macadam (LSCSM). Initially, the mix proportion for LSCSM is determined using the Bailey method. Subsequently, an experimental design based on the response surface method is implemented to comprehensively investigate various properties, including unconfined compressive strength, compressive rebound modulus, flexural strength, and the durability aspects of early drying shrinkage and temperature shrinkage through laboratory experiments. Further, the performance differences between CCSM and LSCSM are analyzed comparatively. The findings reveal that the compressive strength of LSCSM surpasses that of CCSM, albeit with comparatively lower compressive rebound modulus and flexural strength. LSCSM demonstrates a unique blend of characteristics, exhibiting traits of both semi-rigid and flexible materials. Furthermore, LSCSM exhibits favorable crack resistance properties, as evidenced by lower dry shrinkage strain, average dry and temperature shrinkage coefficient compared to CCSM. The proposed LSCSM in this study effectively reduces cement dosage and enhances the crack resistance performance of base materials.

## 1. Introduction

Semi-rigid base materials are widely utilized across various highway grades in China, owing to their commendable strength, stiffness, overall performance, and cost-effectiveness [1,2]. Nevertheless, asphalt pavements situated on semi-rigid base layers often face issues, prominently cracking. More than half of these cracks manifest as reflective cracks [3]. These reflective cracks originate in the base layer and propagate upward due to the influence of temperature fluctuations and vehicular loads following the base layer’s initial cracking. The presence of cracks in the base layer serves as a critical prerequisite for the emergence of reflective cracks. Scholars, through extensive research on semi-rigid base materials, have identified two primary factors contributing to the formation of shrinkage cracks [4,5,6]. The first factor involves stress induced by temperature fluctuations, while the second factor is stress resulting from the reduction in moisture content within the semi-rigid base material. The simultaneous impact of these stress factors is commonly known as the interaction between thermal and dry shrinkage stress. When this cumulative stress reaches the material’s maximum tolerance, it triggers material fracture and the subsequent formation of cracks. Therefore, addressing the improvement of base material crack resistance and reducing occurrences of reflective cracks are crucial for enhancing the quality and lifespan of semi-rigid road surfaces.

Extensive research has explored semi-rigid base materials’ crack resistance performance. In the 1930s, Italy began using geosynthetic materials in road surface structures to prevent reflective crack development [7,8,9]. Meticulous observation and statistical analysis of roads with geosynthetic materials revealed commendable crack resistance in the initial stages of road construction, delaying the onset of reflective cracks. However, over time, crack incidence significantly increased. In the 1950s, France experimented with fly ash and cement mixtures as binders in the base layer, resulting in a reduction in road surface cracks and proving to be more economically viable [10]. The United States conducted numerous studies on fly ash-stabilized macadam mixtures, confirming that fly ash as a binder effectively reduces crack formation [11]. In Canadian regions, asphalt-stabilized crushed stone has been used as a flexible base to mitigate reflective crack effects [12]. In the United States and Austria, a brief curing period of 1 to 3 days followed the completion of semi-rigid base laying and compaction. Vibratory rollers were then used to intentionally induce fine cracking in the base layer, preventing cracks caused by the material’s inherent drying shrinkage and temperature contraction. This proactive measure further reduced the occurrence of reflective cracks in asphalt road surfaces [13,14,15]. In China, the primary focus is on preventive measures against base layer crack formation and expansion. This involves material addition, gradation composition refinement, and precise control of binder dosage [16]. In Germany, France, and Canada, the practice of placing natural 75 mm coarse crushed stones on the road base has been adopted to mitigate road surface crack formation [1,17,18]. When large-sized cement-stabilized macadam is used for the base layer, materials are commonly engineered with a continuous gradation. Conversely, for the sub-base layer, materials usually feature a gap gradation. This strategy has demonstrated effectiveness in averting crack formation. Current research suggests that enhancing the nominal maximum size of aggregates positively influences the crack resistance of cement-stabilized crushed stone materials [19]. Consequently, this measure aids in mitigating the occurrence of reflective cracks on the road surface.

Regarding the early control of shrinkage cracks in base materials, the primary focus has been on researching shrinkage characteristics. However, existing studies have been somewhat limited in exploring the shrinkage performance of large-sized cement-stabilized macadam (LSCSM). This study, therefore, aims to explore the effectiveness of LSCSM when employed as a base material to mitigate surface layer cracking issues induced by dry and temperature-related shrinkage. Initially, the study employs the Bailey method to identify the mix type for LSCSMs and advances to mix proportion design. Utilizing the response surface method, the regression models for mechanical performance responses are developed. Ultimately, the study carries out dry and temperature shrinkage tests to assess and compare the shrinkage performance between LSCSM and conventional cement-stabilized macadam (CCSM). This endeavor furnishes essential experimental data, laying the groundwork for the practical application of LSCSM in the base layer.

## 2. Raw materials and Test Design

### 2.1. Performance Indicators of Raw Materials

#### 2.1.1. Cement

This study employed ordinary Portland cement with a grade of P.O.42.5. The test results for its primary technical indicators are outlined in Table 1. The findings demonstrate that the technical indicators of P.O.42.5 cement align with the standards’ specified requirements.

#### 2.1.2. Coarse and Fine Aggregates

The large-size coarse aggregates and fine aggregates utilized in this study were obtained from Ankang City, Shaanxi Province, China. Following “Test Methods of Aggregate for Highway Engineering” (JTG E42-2005 [20]), various grades of aggregates underwent testing. The pertinent performance test results are outlined in Table 2. The void ratio of the coarse aggregate skeleton under compacted conditions was calculated using Equation (1).

(1) $$VCA=\left(1-\frac{\rho }{\rho_{b}}\right)\times 100$$

Here *VCA* represents the void ratio between coarse aggregate particles in a compacted state, $\rho_{b}$ is the bulk density, and ρ is the accumulated density.

### 2.2. Aggregate Gradation Design

Cement-stabilized macadam materials are typically designed using two types of gradation: continuous gradation and gap gradation. The current theories referenced for gradation design mainly include the maximum density curve and the gradual filling theory [21]. In the design concept of large-size cement-stabilized macadam (LSCSM), a crucial aspect is to achieve a closely interlocked skeleton of LSCSM. The main idea of the gradual filling theory is to use smaller-sized aggregates to fill the voids in the skeleton created by interlocking larger-sized aggregates. This design method enhances the interlocking impact of the skeleton in the mixture. Currently, the Bailey method stands as a relatively comprehensive theory and method for skeleton compact gradation design both domestically and internationally. In this study, the gradation design for LSCSM was conducted based on the Bailey method. The separation sieve sizes for coarse and fine aggregates are determined according to Equation (2).

(2) PCS=NMPS×0.22

Here, PCS represents the control particle size of the aggregate, while NMPS stands for the nominal maximum size of the aggregate.

**Figure 1:**
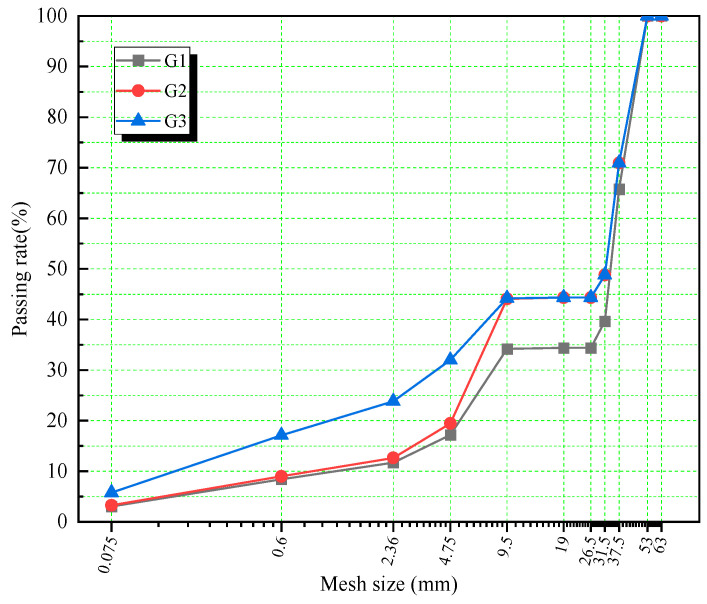
Design gradation curve.

In this study, the nominal maximum size of the LSCSM was 53 mm, and the boundary particle size between coarse and fine aggregates was 13.2 mm. To prevent the impact and damage caused by the interlocking state of fine aggregate skeleton particles, it was proposed that the particle distribution within the range of 13.2 mm to 31.5 mm be excluded from the gradation. Following the design principles of the Bailey method, the ratio of 0~5 mm, 5~10 mm, and 30~50 mm in the mixture of LSCSM was established as 0.14:0.20:0.66. To assess the influence of gradation on the properties of cement-stabilized macadam, the proportions of coarse and fine aggregates were adjusted, leading to the formulation of three distinct gradations. The proportions for each grade of aggregates are shown in Table 3 and Figure 1.

Different gradations of cement-stabilized macadam have different void space. For better experimental design, this study characterizes different gradations by void space. The void is the part of the cement and fine aggregate slurry remaining after filling the void of the coarse aggregate skeleton, which consists of three parts: connected void, semi-connected void, and closed void, and the sum of the three is the void space. The void ratio is the percentage of the total void space to the total mix volume. In this study, the void ratio of cement-stabilized macadam was examined using the weighing in water method. G1, G2, and G3 have a void space of 32%, 25%, and 18%, respectively.

### 2.3. Test Design

The response surface methodology primarily involves orthogonal designs of quadratic regression for given influencing factors [22,23]. Through combinations of experiments at different levels of these factors, the methodology assesses how the response values change under varying conditions. This study considers cement dosage, gradation type (void ratio), and curing period as the three influencing factors for the response surface methodology. Specifically, cement dosages of 2%, 3%, and 4% have been selected. Gradation types, identified in Section 2.2, have been designated as factors 1, 2, and 3. It is worth noting that the gradation type is correlated with the void ratio. Curing periods were chosen as 7 days, 14 days, and 21 days in length. Table 4 delineates the levels and design of the influencing factors.

**Figure 2:**
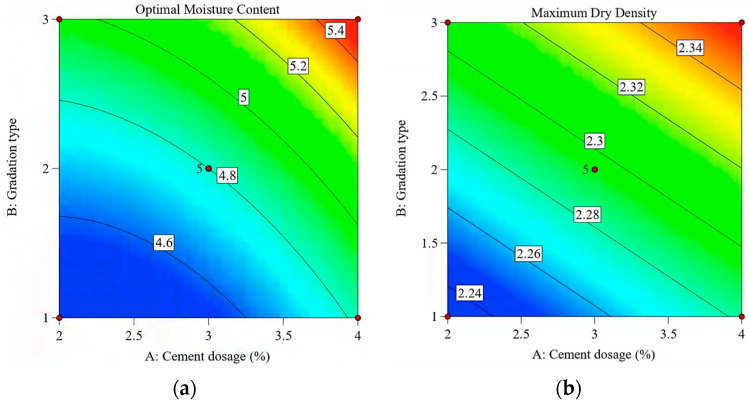
Compaction test results for (**a**) optimal moisture content and (**b**) maximum dry density.

The experimental design was conducted using the Box–Behnken design method, generating a three-factor, three-level experimental design table using Design Expert 13.0 software. The response indicators included compressive strength, compressive rebound modulus, and flexural strength. To determine the LSCSM’s optimal moisture content and maximum dry density, vibrating compaction tests were conducted on the LSCSM. In accordance with the vibrating compaction test method outlined in the specifications, the maximum dry density and optimum moisture content of LSCSM were determined [24]. The test results under various conditions are presented in Table 5 and Figure 2.

**Figure 3:**
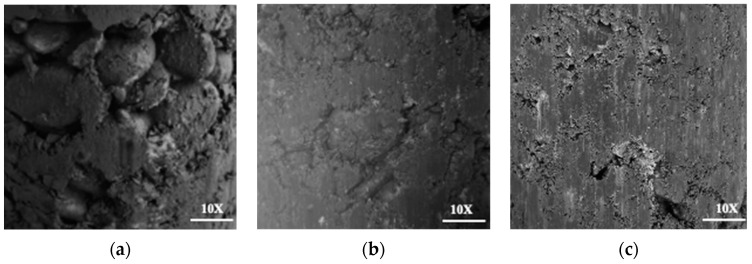
Specimen section shape in (**a**) G1, (**b**) G2, (**c**) G3.

It was found that under the three gradation conditions, as the cement dosage increased, both the maximum dry density and optimum moisture content increased. G1 has the highest coarse aggregate content, while G3 has the highest fine aggregate content. Based on the experimental results, it can be inferred that the specimen in G1 represented a porous structure, the specimen in G2 approached a skeleton interlocking structure, and the specimen in G3 approached a suspended compact structure. Figure 3 shows the specimen section shape under different gradations. It can also be seen from the figure that there was a great difference in the void fraction of the specimens prepared from the three gradations.

## 3. Test Methods

### 3.1. Compressive Strength

According to the unconfined compressive strength test method outlined in the specifications, a custom-made Φ200 mm × 200 mm cylindrical mold was used in this study [24]. The compressive strength tests were conducted using a YZM-A road material strength tester. The experimental design detailed in Table 5 was adhered to, where specimens were formed using the vibrating compaction method. A day prior to the designated curing period, the specimens were immersed in water for a duration of 24 h. Subsequently, the compressive strength test was conducted using a universal testing machine after the curing period. Thirteen repetitions of the test were performed for each set of conditions. The compressive strength was calculated according to Equations (3) and (4).

(3) $$R_{c}=\frac{P}{A}$$

(4) $$A=\frac{1}{4}\pi D^{2}$$

Here, *R_c_* represents the compressive strength, *P* denotes the maximum pressure at the time of specimen failure, *A* signifies the cross-sectional area of the specimen, and *D* is the diameter of the specimen.

### 3.2. Compressive Rebound Modulus

The modulus tests were conducted following the specifications [24]. Universal testing machine equipment was used to conduct the test. The specimen preparation process for the rebound modulus test is identical to the specimen preparation process for the compressive strength test. Equation (5) for calculating the rebound modulus is as follows.

(5) $$E_{c}=\frac{ph}{l}$$

where, *E_c_* represents the compressive rebound modulus, *p* stands for the unit pressure, *h* denotes the height of the specimen, and *l* signifies the rebound deformation of the specimen.

### 3.3. Flexural Strength

The flexural strength of LSCSM was determined using the three-point bending test method for inorganically bound stabilized materials according to the specifications in [24]. Universal testing machine equipment was used to conduct the test. The test specimen dimensions were 150 mm × 150 mm × 550 mm. The loading rate was set at 50 mm/min, and loading continued until the specimens experienced fracture failure. The ultimate failure load was recorded, and the formula for calculating flexural strength is given by Equation (6).

(6) $$R_{s}=\frac{PL}{b^{2}h}$$

Here, *R_s_* is the flexural strength, *P* represents the failure load, and *L* denotes the distance between the two support points, while *h* and *b* represent the height and width of the specimen, respectively.

### 3.4. Shrinkage Test

#### 3.4.1. Dry Shrinkage Test

According to the specifications, the beam specimen dimensions were selected as 150 mm × 150 mm × 550 mm [24]. The shrinkage tester employed a Tianshu Xing brand TD566 dry shrinkage tester. The specimens were prepared using the vibration molding method and demolded after 6 h. The calculation methods for water loss rate, dry shrinkage strain, dry shrinkage coefficient are shown in Equations (7)–(10).

(7) $$\omega_{i}=\left(m_{i}-m_{i+1}\right)/m_{p}$$

(8) $$\delta_{i}=\left(\sum_{j=1}^{4}X_{i,j}-\sum_{j=1}^{4}X_{i+1,j}\right)/2$$

(9) $$\varepsilon_{i}=\delta_{i}/l$$

(10) $$\alpha_{di}=\varepsilon_{i}/\omega_{i}$$

Here, $\omega_{i}$ is the *i*-th time water loss rate, $m_{i}$ is the mass of the standard specimen measured in the *i*-th test, $m_{p}$ is the constant mass after drying the standard specimen, $\delta_{i}$ is the *i*-th time observed dry shrinkage, $X_{i,j}$ is the reading of the *j*-th dial indicator in the *i*-th test, $\varepsilon_{i}$ represents the *i*-th dry shrinkage strain, *l* denotes the length of the standard specimen, and $\alpha_{di}$ is the *i*-th dry shrinkage coefficient.

#### 3.4.2. Temperature Shrinkage Test

The temperature shrinkage test was conducted following the specification [24]. The high and low temperature alternating box was a Shanghai Yiheng BPH-060A high and low temperature test box. The calculation method for the temperature shrinkage coefficient is shown in Equations (11) and (12).

(11) $$\varepsilon_{i}=\frac{l_{i}-l_{i+1}}{L_{0}}$$

(12) $$\alpha_{t}=\frac{\varepsilon_{i}}{t_{i}-t_{i+1}}$$

Here, $\varepsilon_{i}$ is the average shrinkage strain of the *i*-th temperature zone, $l_{i}$ is the average shrinkage of the i-th temperature zone, $L_{0}$ is the initial length of the specimen, $\alpha_{t}$ is the coefficient of thermal contraction, $t_{i}$ is the *i*-th temperature zone.

## 4. Results Analysis

### 4.1. Statistical Analysis

The mechanical tests were conducted based on the response surface experimental design, and the results are presented in Table 5. Design-Expert 13 software was employed for an in-depth analysis of the correlation among compressive strength, compressive rebound modulus, and flexural strength, considering three influencing factors: cement content, gradation type (void ratio), and curing period. Following the principles of response surface design, quadratic models were established using the least squares method to depict relationships between compressive strength, compressive rebound modulus, and flexural strength as response variables, as illustrated in Equations (13)–(15).

(13) $$R_{c}=-4.37+4.27A+0.817B+0.597429C-0.4905A^{2}-0.4905B^{2}-0.01C^{2}$$

(14) $$\begin{array}{ll} E_{c}= & 1326.875-25.275A-126.975B-142.08571C+10AB+15.96429AC\\ & +27.67857BC-18.6A^{2}-32.6B^{2}+2.79898C^{2} \end{array}$$

(15) $$\begin{array}{ll} R_{s}= & 0.81875+0.01175A-0.08175B-0.091857C+0.005AB\\ & +0.009643AC+0.0175BC-0.0155A^{2}-0.0205B^{2}+0.001878C^{2} \end{array}$$

Here, *R_c_* represents the compressive strength; *E_c_* is the compressive rebound modulus; *R_s_* represents the flexural strength; *A*, *B*, and *C* represent the cement content, gradation type (void ratio), and curing period, respectively. It is worth noting that the gradation type is correlated with the void ratio.

To assess the accuracy and reliability of the regression models, variance analysis and statistical analysis were conducted to test the significance of the model error sources. The results of the variance analysis and statistical analysis are presented in Table 6 and Table 7. The significance of the regression models was determined using both *F*-values and *p*-values. From Table 6, it can be observed that the *F*-values for the compressive strength, compressive rebound modulus, and flexural strength models are 12.38, 18.71, and 13.96, respectively, with corresponding *p*-values of 0.0016, 0.0004, and 0.0011. Generally, *p*-values less than 0.0001 are deemed highly significant and values falling between 0.0001 and 0.05 are considered significant, while values greater than 0.05 are regarded as not significant.

The established models exhibit extremely high significance in explaining the relationship between the response target values and the quadratic polynomial regression models. They effectively interpret the functional relationship between the independent variables and the response target values with statistical significance. Additionally, the lack-of-fit results show that the models corresponding to the three response values are not significant, further indicating high fitting accuracy and strong reliability. Therefore, using the established models for fitting analysis of experimental results is feasible. The goodness of fit (*R*^2^) of the models explains the degree of difference between the response values and actual values. *R*^2^ ranges from 0 to 1, and the closer its value is to 1, the closer the model’s test values are to the predicted values, indicating greater reliability. The model correlation coefficients *R*^2^ are 0.9409, 0.9601, and 0.9472, and the adjusted correlation coefficients are 0.8649, 0.9088, and 0.8794, respectively. This result indicates high fitting accuracy and strong reliability of the selected models.

**Figure 4:**
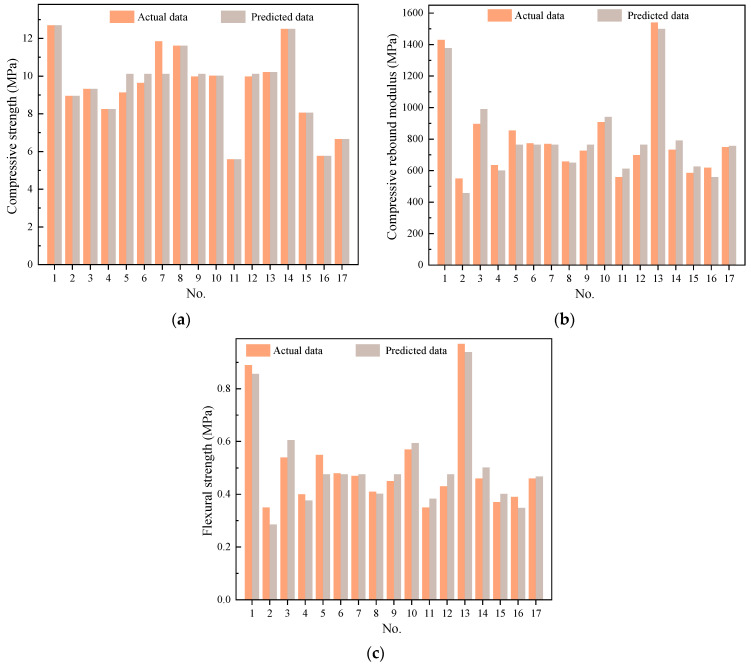
The relationship between test and predicted value for (**a**) compressive strength, (**b**) compressive rebound modulus, and (**c**) flexural strength.

The effectiveness of the data and models was further diagnosed using the data monitoring feature of Design-Expert 13. The histogram diagrams were created with compressive strength, compressive rebound modulus, and flexural strength test results as well as model-predicted values as the *x* and *y* coordinates, as shown in Figure 4. It is evident that the data points are roughly distributed along a straight line and are close to the *y* = *x* line. This indicates a good fit of the regression models, allowing for the analysis and prediction of compressive strength, compressive rebound modulus, and flexural strength of LSCSM.

### 4.2. Performance Responses Based on the Regression Models

#### 4.2.1. Compressive Strength

**Figure 5:**
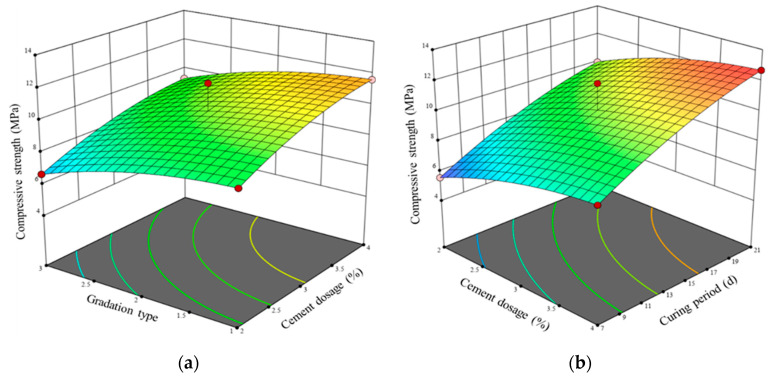

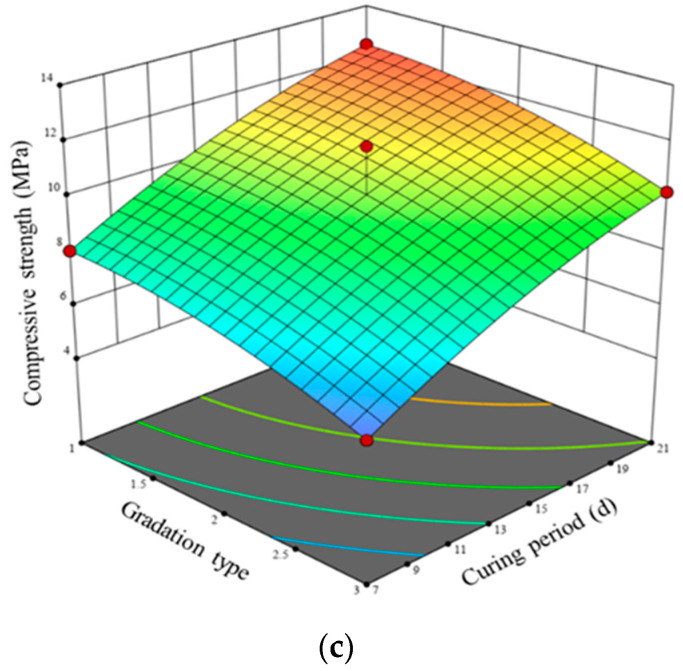
Effect of difference factors on compressive strength (**a**) gradation type and cement dosage, (**b**) cement dosage and curing period, (**c**) gradation type and curing period.

Table 5 highlights the substantial impact of cement content, gradation type (void ratio), and curing period on the compressive strength of LSCSM. To visually discern the impact trend of interaction effects among factors on the response, three-dimensional response surface graphs were constructed, utilizing cement content, gradation type (void ratio), and curing period as the *x* and *y* axes and compressive strength as the *z* axis, as shown in Figure 5. The steeper the response surface, the more pronounced the interaction effects among factors. Conversely, a shallower response surface indicates less substantial interaction effects. The compressive strength of LSCSM exhibits an increase with rising cement content and curing period. Moreover, coarser gradation correlates with higher compressive strength in LSCSM. In these scenarios, the curve representing the curing period exhibits a steeper incline compared to the curve of the cement content, and the cement content curve is steeper than that of the gradation type. Regarding G1, when the cement content rises from 2% to 4%, the compressive strength at 7 days is elevated from 6.22 MPa to 8.88 MPa. At a cement content of 3%, the compressive strength at 7 days for G1, G2, and G3 is 8.04 MPa, 7.39 MPa, and 5.75 MPa, respectively. For a cement content of 4%, using G1, the compressive strength at 7 days, 14 days, and 28 days is 8.88 MPa, 11.59 MPa, and 14.07 MPa, respectively. Comparison of the compressive strengths of different gradations shows that G1 has the highest compressive strength. This is due to the fact that the strength of the LSCSM mainly arises from the interlocking action between aggregates. The addition of fine aggregates in G1 reduces the interlocking effect or increases the void ratio, leading to a decrease in strength. This indicates that compactness has a significant impact on compressive strength. On the other hand, when the results of compressive strength tests at 7 days and 21 days are compared, the strength growth with curing period is smaller for G1 than for G3. G3 has a smaller void ratio than G1. This could be attributed to the increased amount of fine aggregates in G3, resulting in more complete hydration reactions as the curing period progresses.

#### 4.2.2. Compressive Rebound Modulus

**Figure 6:**
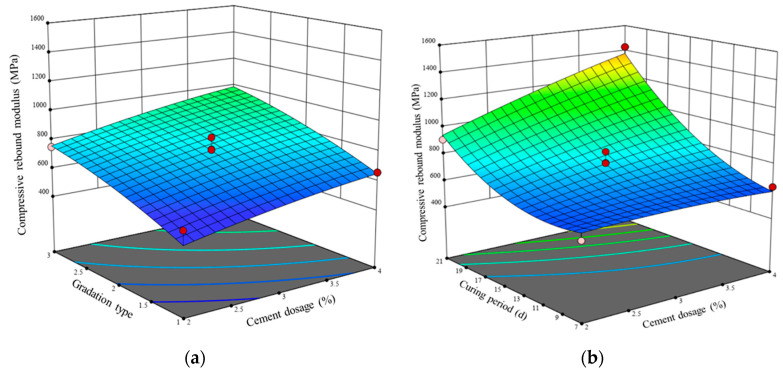

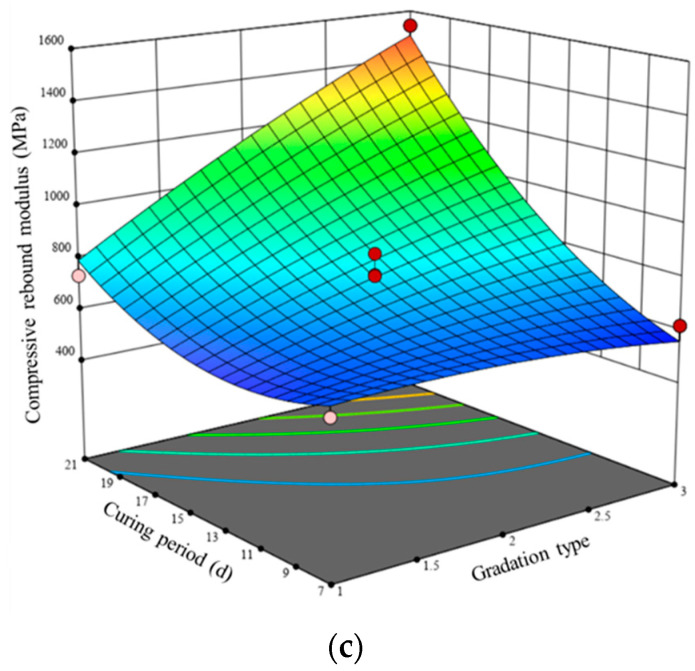
Effect of difference factors on compressive rebound modulus (**a**) gradation type and cement dosage, (**b**) cement dosage and curing period, (**c**) gradation type and curing period.

Using cement content, gradation type, and curing period as the *x* and *y* axes and the compressive rebound modulus as the *z* axis, a three-dimensional response surface graph was constructed, as shown in Figure 6. Analyzing the experimental results in conjunction with the regression model, when the cement content is 4% and G1 is used, the compressive rebound modulus of the LSCSM at 7 days, 14 days, and 28 days is 591 MPa, 650 MPa, and 1587 MPa, respectively. For G1, at 28 days, the compressive rebound modulus of LSCSM with 2%, 3%, and 4% cement content is 947 MPa, 1286 MPa, and 1587 MPa, respectively. When the cement content is 2%, the compressive rebound modulus of LSCSM at 28 days for G1, G2, and G3 is 947 MPa, 1517 MPa, and 2022 MPa, respectively. From the above results, it can be observed that the compressive rebound modulus significantly increases with the extension of the curing period. The addition of cement content increases the compressive rebound modulus of LSCSM, but the increase is relatively small. In terms of gradation type, an increase in fine aggregate content leads to a larger compressive rebound modulus. When considering the compressive rebound modulus results, it is evident that the compressive rebound modulus of LSCSM is lower than that of conventional cement-stabilized macadam materials, while it is higher than the modulus of flexible materials such as graded gravel. Therefore, LSCSM exhibits characteristics of both semi-rigid and flexible materials.

#### 4.2.3. Flexural Strength

**Figure 7:**
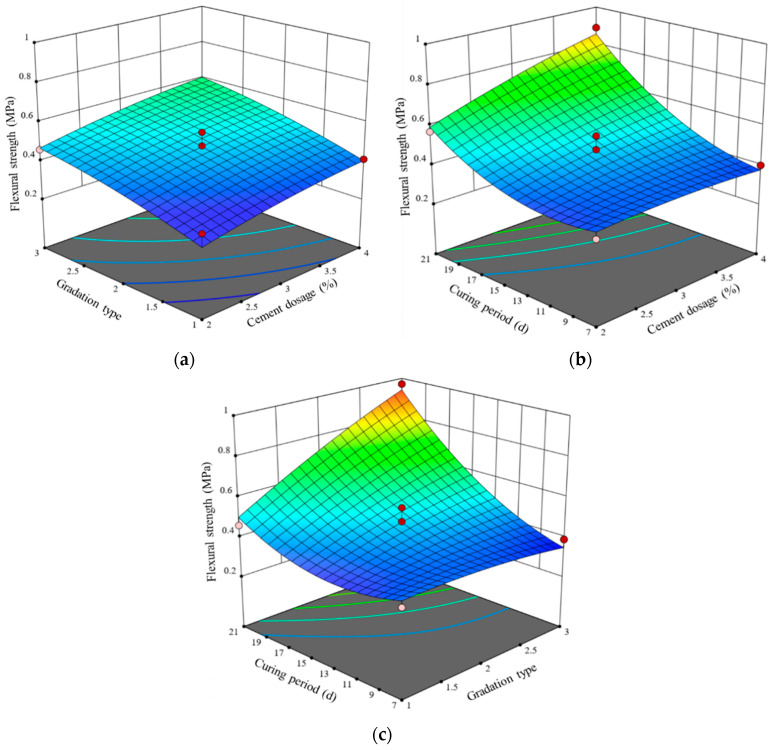
Effect of difference factors on flexural strength (**a**) gradation type and cement dosage, (**b**) cement dosage and curing period, (**c**) gradation type and curing period.

Using cement content, gradation type, and curing period as the *x* and *y* axes and flexural strength as the *z* axis, a three-dimensional response surface graph is constructed, as shown in Figure 7. Analyzing the experimental results in conjunction with the regression model, when the cement content is 4% and G2 is used, the flexural strength of the LSCSM at 7 days, 14 days, and 28 days is 0.37 MPa, 0.52 MPa, and 1.37 MPa, respectively. With G2, at 28 days, the flexural strength for 2%, 3%, and 4% cement content in the LSCSM is 0.97 MPa, 1.19 MPa, and 1.37 MPa, respectively. When the cement content is 3%, the flexural strength at 28 days for G1, G2, and G3 in the LSCSM is 0.82 MPa, 1.19 MPa, and 1.51 MPa, respectively. It can be noticed that as the curing period increases, the flexural strength of the three gradations of LSCSM continues to rise. This is attributed to the ongoing reactions and continuous solidification and hardening of the cementitious material during the curing process, resulting in the development of structural strength. The bonding strength steadily increases with the prolonged curing period. As the reactions approach completion, the overall strength of the material gradually stabilizes. A comparison of the flexural strength among different gradations reveals that G1 exhibits lower flexural strength. This can be attributed to the interface transition zone between the coarse aggregates forming the skeleton and the filling materials, characterized by very low strength and relatively weak bonding forces, making it the structurally weakest component. With an increase in the size of the coarse crushed stone, the interface transition zone expands, leading to significant stress concentration at the defect edges. This, in turn, results in a diminished load-bearing capacity of this area and a subsequent reduction in flexural strength.

### 4.3. Optimization and Comparative Analysis of LSCSM

#### 4.3.1. Comparative Analysis of Mechanical Properties

Following the analysis of variance and response surface analysis, the cement dosage and gradation type of LSCSM have been systematically optimized using the principles of response surface methodology. The optimal cement dosage has been determined as 3.5%, with G2 being the selected gradation type. Under these optimized conditions, the predicted values for the 7-day compressive strength, compressive rebound modulus, and flexural strength of LSCSM based on the regression model, are 7.93 MPa, 617 MPa, and 0.39 MPa, respectively.

**Figure 8:**
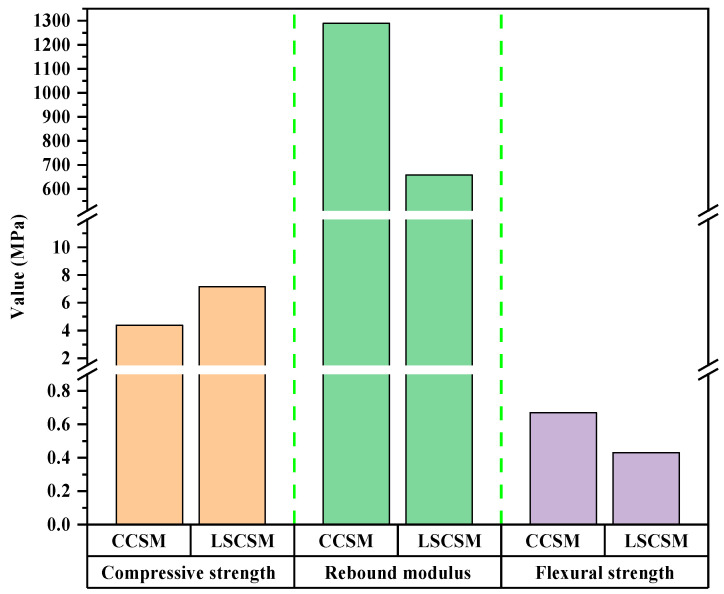
The comparison results between CCSM and LSCSM.

To compare the difference in performance characteristics between LSCSM and conventional cement-stabilized macadam (CCSM), both specimens were molded in this study. Among them, the gradation of LSCSM and CCSM is shown in Table 8. The cement content is selected as 3.5%. The comparative results of the mechanical properties between LSCSM and CCSM are illustrated in Figure 8. The analysis of these results clearly indicates that the compressive strength of LSCSM is 1.6 times that of CCSM. The 7-day compressive rebound modulus of LSCSM is approximately 49% lower than that of CCSM, and the flexural strength of LSCSM at 7 days is only about 60% of that of CCSM.

#### 4.3.2. Comparative Analysis of Dry Shrinkage Properties

**Figure 9:**
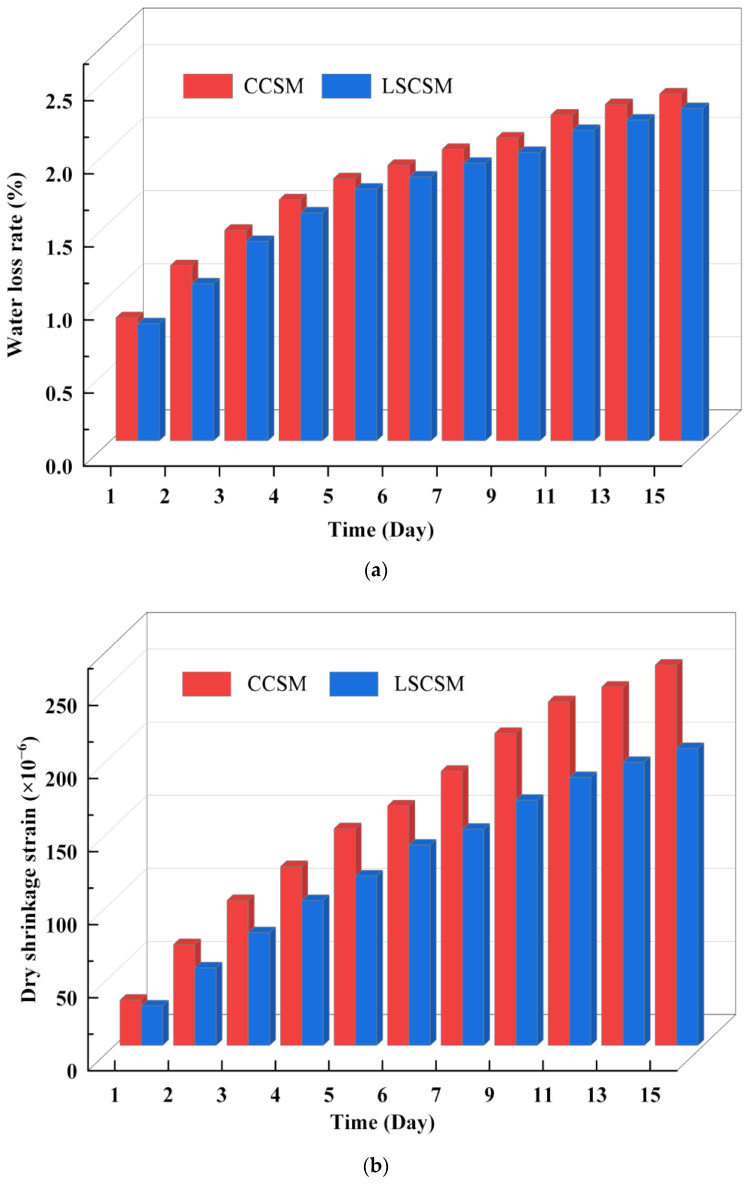

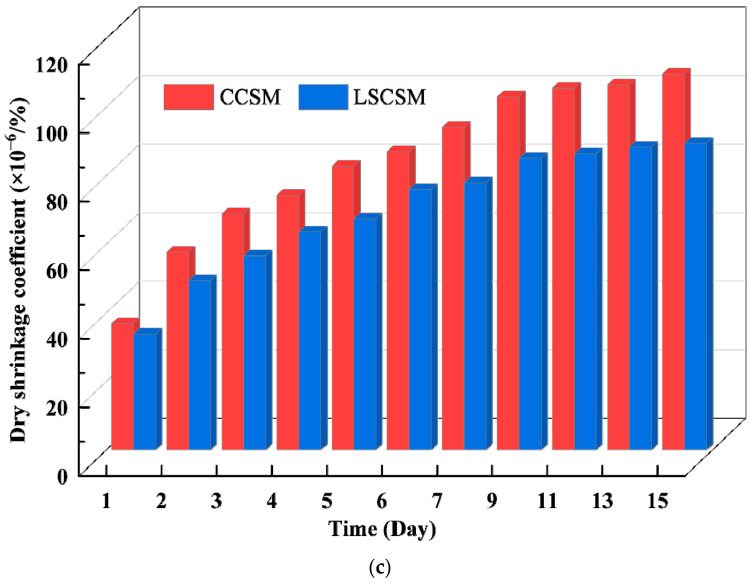
The different materials’ variation curve: (**a**) water loss rate, (**b**) dry shrinkage strain, (**c**) dry shrinkage coefficient.

The results of the dry shrinkage experiments for LSCSM and CCSM are shown in Figure 9. The curves depict the trends of water loss rate, dry shrinkage strain, and dry shrinkage coefficient with the increase in curing period for both materials. The experimental results are analyzed to summarize the patterns of water loss rate and shrinkage strain during the dry shrinkage process for both materials.

From the results of water loss rate, CCSM exhibits a higher water loss rate than LSCSM. This is attributed to the higher proportion of coarse crushed stone in LSCSM, resulting in a lower proportion of water-stable fines filling the voids. In comparison to CCSM, LSCSM absorbs less moisture during the curing period, leading to a lower overall moisture content in the specimens. In the dry shrinkage test, due to the inherently lower moisture content in the entire material structure, moisture is less prone to dissipate, resulting in a lower water loss rate for LSCSM.

From the results of the dry shrinkage strain, it can be observed that with the increase in curing period, the dry shrinkage strain of both materials increases, but the rate of increase gradually slows down. Under the same curing conditions, the dry shrinkage strain of LSCSM is smaller than that of CCSM. On the first day, the dry shrinkage strain of LSCSM is 12.69% less than that of CCSM. From the 2nd day to the 15th day, compared to CCSM, the dry shrinkage strain of LSCSM is reduced by 16% to 23%. This result indicates that LSCSM effectively enhances the resistance of the base material to dry shrinkage. Simultaneously, the lower dry shrinkage strain can effectively reduce the internal stress generated by shrinkage in the base structure, thereby alleviating the formation and expansion of dry shrinkage cracks.

From the results of the dry shrinkage coefficient calculations, it can be observed that the dry shrinkage coefficients of both materials continuously increase with curing period, but the rate of increase gradually slows down. Comparing the dry shrinkage coefficients of the two materials at the same time, it is found that the dry shrinkage coefficient of LSCSM is smaller than that of CCSM material. Specifically, at 3 days, 7 days, and 15 days, the dry shrinkage coefficients of LSCSM are 17.82%, 17.20%, and 18.42% less than those of CCSM, respectively. The relationship between the dry shrinkage coefficient and curing period can be well established using a logarithmic function with high fitting accuracy. The predicted results indicate that the dry shrinkage coefficient of the material gradually becomes a constant value in the later stages of curing. A smaller dry shrinkage coefficient indicates that the base material is less sensitive to dry shrinkage deformation. This suggests that, compared to CCSM, LSCSM exhibits smaller shrinkage deformations under the same dry conditions, demonstrating excellent resistance to dry shrinkage. It also implies that LSCSM has a certain effect in delaying the formation of reflective cracks on the asphalt surface.

**Figure 10:**
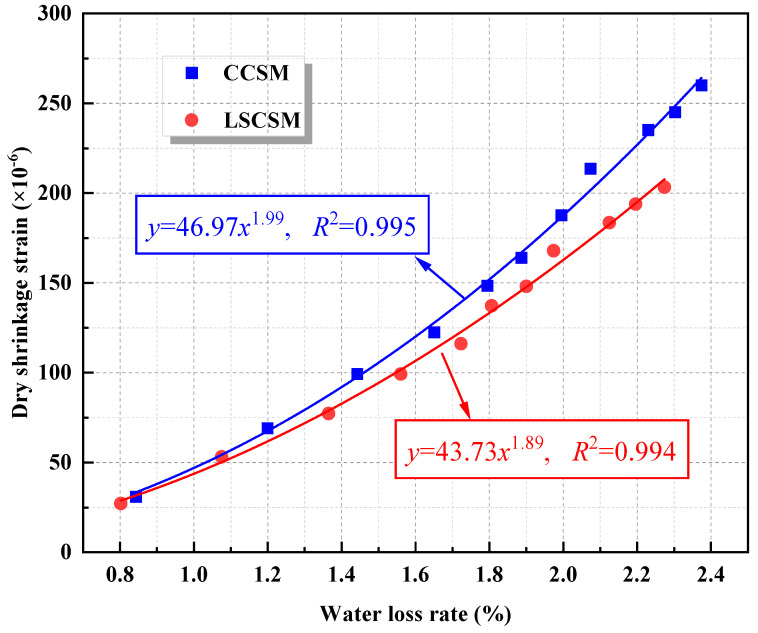
The relationship between water loss rate and dry shrinkage strain.

To further investigate the shrinkage performance of the base material, this study established the correlation model between the water loss rate and dry shrinkage strain. Figure 10 illustrates that the dry shrinkage strain of both materials increases with a rise in the water loss rate. The relationship between them follows a power function. The fitting results indicate that the growth rate of dry shrinkage strain for LSCSM is smaller than that for CCSM. Specifically, under the same water loss rate, the dry shrinkage strain of CCSM is approximately 1.2 times that of LSCSM. Considering the water loss rate results, it is evident that CCSM experiences a higher water loss rate compared to LSCSM, resulting in a notable difference in dry shrinkage strain between the two materials. In the semi-rigid base asphalt pavement, the construction of the cement-stabilized macadam base layer leads to a continual reduction of moisture due to capillary tension and the forces between adsorbed water molecules, causing an increase in dry shrinkage stress. In the early stages of construction, rapid water evaporation induces significant dry shrinkage stress. However, the tensile strength of the material has not yet reached its maximum, making it more susceptible to dry shrinkage cracks. If the asphalt surface layer is not promptly placed after the curing period of the cement-stabilized macadam base layer, the extended exposure time to air intensifies the development of dry shrinkage cracks. The results of the dry shrinkage test affirm that LSCSM exhibits superior anti-shrinkage performance compared to CCSM.

#### 4.3.3. Comparative Analysis of Temperature Shrinkage Properties

**Figure 11:**
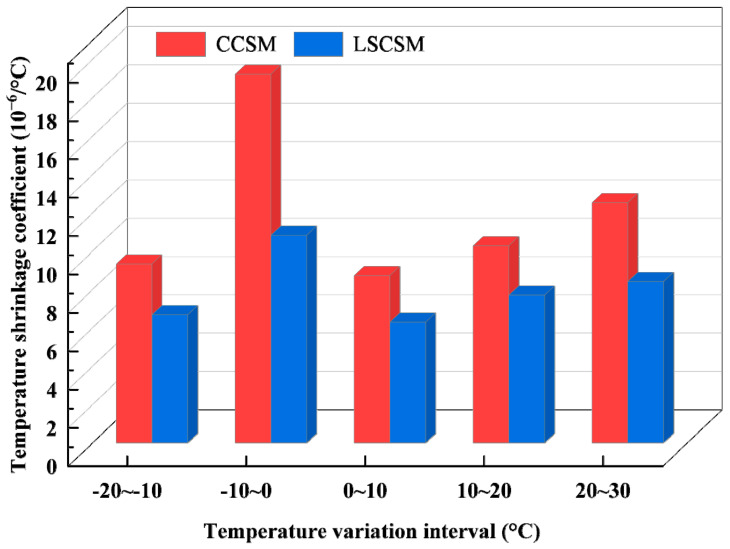
Temperature shrinkage coefficient variation trend.

The temperature shrinkage coefficient and trends of LSCSM and CCSM in each temperature interval are illustrated in Figure 11.

The analysis of Figure 11 indicates that, within three consecutive temperature intervals from 30 °C to 0 °C, the temperature shrinkage coefficients of both materials decrease as the temperature drops, showing a relatively gradual reduction trend. In the temperature range of 0 °C to −10 °C, the temperature shrinkage coefficient increases significantly compared to the temperature range of 30 °C to 0 °C. As the temperature further decreases to the range of −10 °C to −20 °C, the temperature shrinkage coefficient decreases. Moreover, the temperature shrinkage coefficients in the temperature range of −10 °C to −20 °C are similar to those in the range of 0 °C to 10 °C. The experimental results indicate that the temperature shrinkage coefficients of both materials vary more drastically in the temperature range of 0 °C to −20 °C than in the range of 30 °C to 0 °C. This implies that the temperature shrinkage performance of the materials is more unfavorable, especially under low-temperature conditions, and is more prone to thermal contraction cracks, particularly in the range of 0 °C to −10 °C.

**Figure 12:**
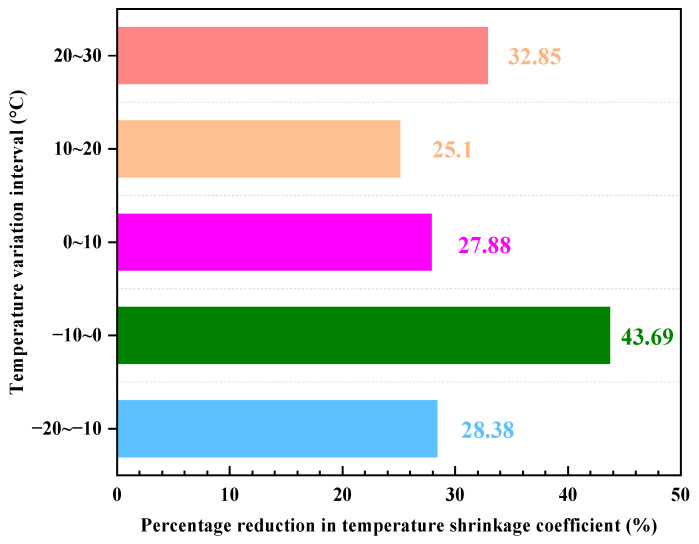
Percentage reduction in temperature shrinkage coefficient.

The comparison of the temperature shrinkage coefficients of the two materials in the same temperature range reveals, as shown in Figure 12, that the temperature shrinkage coefficient of specimens of LSCSM is significantly smaller than that of CCSM. This difference is most pronounced in the temperature range of 0 °C to −10 °C. In the 0 °C to −10 °C temperature range, the temperature shrinkage coefficient of CCSM is 19.205 × 10^−6^/°C, while the temperature shrinkage coefficient of LSCSM is 10.815 × 10^−6^/°C. Compared to CCSM, its temperature shrinkage coefficient has decreased by 43.69%. In the other four temperature ranges, the temperature shrinkage coefficient has decreased by 25% to 33%. From the experimental results, it can be observed that under the same temperature conditions, the base material of LSCSM exhibits smaller temperature shrinkage deformation compared to CCSM. In other words, the material shows a significant reduction in temperature shrinkage stress and is less prone to cracking due to excessive shrinkage, demonstrating excellent resistance to temperature shrinkage.

## 5. Conclusions

The study conducted a comprehensive assessment of the mechanical and shrinkage properties of large particle size cement-stabilized macadam (LSCSM), a novel type of base material. Firstly, utilizing the response surface method, the investigation analyzed the influence of cement dosage, gradation type (void ratio), and curing period on the compressive strength, compressive rebound modulus, and flexural strength of LSCSM. Subsequently, considering the determined mechanical properties, appropriate cement dosage and gradation type (void ratio) were identified, facilitating a comparative analysis of the mechanical and shrinkage characteristics between LSCSM and conventional cement-stabilized macadam (CCSM). The main conclusions are as follows.

1. Quadratic regression models were developed for cement dosage, gradation type, curing period, and the corresponding compressive strength, compressive rebound modulus, and flexural strength using the Box–Behnken design. The model correlation coefficients *R*^2^ are 0.9409, 0.9601, and 0.9472, respectively. Additionally, the adjusted correlation coefficients are 0.8649, 0.9088, and 0.8794, respectively. The results affirm the high precision and reliability in model fitting.
2. A comparison of the mechanical properties between CCSM and LSCSM indicates that the compressive strength of the LSCSM surpasses that of CCSM. Specifically, the 7-day compressive rebound modulus of LSCSM is approximately 49% lower than that of CCSM, and the 7-day flexural strength of LSCSM is only around 60% of that of CCSM.
3. In comparison with CCSM, the dry shrinkage strain and dry shrinkage coefficient of LSCSM decreased by 21.86% and 18.49%, respectively, at 15 days. Although the temperature shrinkage pattern of LSCSM generally resembled that of CCSM, a notable reduction in the temperature shrinkage coefficient within the same temperature range was observed. LSCSM exhibits effective capabilities in mitigating the occurrence of shrinkage cracks and preventing the development of reflective cracks in asphalt pavement.
4. The shrinkage and the measured properties have direct relation with the void space in designed graded aggregates. Establishing a link between void fraction and the shrinkages and properties of cement-stabilized macadam can help to understand the effect of void ration on properties. In further, it can attempt to establish the relationship between the voids with shrinkages and properties.

## Figures and Tables

**Table 1:** Test results of the technical indicators for cement.

Technical Indicators	Test Results	
Specific surface area (m^2^/kg)	338	
Fineness (%)	4.1	
Setting time (min)	Initial time	308
	Final time	402
Soundness	1.6	
Compressive strength (MPa)	3 d	22.5
	28 d	45.3
Flexural strength (MPa)	3 d	5.2
	28 d	8.4

**Table 2:** Basic performance results of aggregates.

Aggregate Sizes	Accumulated Density (g/cm^3^)	Solidification Density (g/cm^3^)	Bulk Density (g/cm^3^)	Apparent Density (g/cm^3^)	Percentage of Voids in Aggregate (%)
30~50 mm	1.653	1.705	2.669	2.675	38.06
5~10 mm	1.648	1.688	2.629	2.638	37.31
0~5 mm	1.605	1.674	2.613	2.616	-

**Table 3:** Design gradation.

Aggregate Size	Gradation 1 (G1)	Gradation 2 (G2)	Gradation 3 (G3)
0~5 mm	14	15	30
5~10 mm	20	29	14
30~50 mm	66	56	56

**Table 4:** Levels and design of influencing factors.

Factors	Number	Level		
		−1	0	1
Cement dosage	A	2	3	4
Gradation type	B	1	2	3
Curing period	C	7	14	21

**Table 5:** Experimental design and responses.

No.	Factor			Test Conditions		Responses		
	Cement Dosage (%)	Gradation Type	Curing Period (d)	Optimal Moisture Content (%)	Maximum Dry Density (g/cm^3^)	Compressive Strength (MPa)	Compressive Rebound Modulus (MPa)	Flexural Strength (MPa)
1	4	2	21	5.2	2.32	12.69	1430	0.89
2	2	1	14	4.5	2.24	8.95	549	0.35
3	4	3	14	5.5	2.36	9.32	897	0.54
4	4	2	7	5.2	2.32	8.25	634	0.4
5	3	2	14	4.8	2.29	9.13	855	0.55
6	3	2	14	4.8	2.29	9.64	773	0.48
7	3	2	14	4.8	2.29	11.85	769	0.47
8	4	1	14	4.8	2.28	11.61	657	0.41
9	3	2	14	4.8	2.29	9.98	726	0.45
10	2	2	21	4.7	2.27	10.02	908	0.57
11	2	2	7	4.7	2.27	5.58	559	0.35
12	3	2	14	4.8	2.29	9.98	698	0.43
13	3	3	21	5.2	2.34	10.21	1540	0.97
14	3	1	21	4.6	2.26	12.5	732	0.46
15	3	1	7	4.6	2.26	8.06	585	0.37
16	3	3	7	5.2	2.34	5.77	618	0.39
17	2	3	14	5.0	2.30	6.66	749	0.46

**Table 6:** Analysis of the variance for the mechanical performance results.

Factor	Sum of Squares	Degrees of Freedom	Mean Square	*F*-Value	*p*-Value	
Compressive strength (MPa)						
Quadratic model	67.52	9	7.5	12.38	0.0016	Significant
A-Cement dosage	14.2	1	14.2	23.44	0.0019	
B-Gradation type (Void ratio)	10.49	1	10.49	17.3	0.0042	
C-Curing period	39.43	1	39.43	65.05	<0.0001	
AB	0	1	0	0	1	
AC	0	1	0	0	1	
BC	0	1	0	0	1	
A^2^	1.01	1	1.01	1.67	0.2371	
B^2^	1.01	1	1.01	1.67	0.2371	
C^2^	1.01	1	1.01	1.67	0.2371	
Compressive rebound modulus (MPa)						
Quadratic model	1.192 × 10^6^	9	132400	18.71	0.0004	Significant
A-Cement dosage	90,951.13	1	90,951.13	12.85	0.0089	
B-Gradation type (Void ratio)	2.051 × 10^5^	1	205,100	28.98	0.001	
C-Curing period	6.127 × 10^5^	1	612,700	86.57	<0.0001	
AB	400	1	400	0.0565	0.8189	
AC	49,952.25	1	49,952.25	7.06	0.0326	
BC	1.502 × 10^5^	1	150,200	21.22	0.0025	
A^2^	1456.67	1	1456.67	0.2058	0.6638	
B^2^	4474.78	1	4474.78	0.6322	0.4526	
C^2^	79,200.52	1	79,200.52	11.19	0.0123	
Flexural strength (MPa)						
Quadratic model	0.4603	9	0.0511	13.96	0.0011	Significant
A-Cement dosage	0.0325	1	0.0325	8.87	0.0205	
B-Gradation type (Void ratio)	0.0741	1	0.0741	20.23	0.0028	
C-Curing period	0.238	1	0.238	64.98	<0.0001	
AB	0.0001	1	0.0001	0.0273	0.8734	
AC	0.0182	1	0.0182	4.97	0.0609	
BC	0.06	1	0.06	16.38	0.0049	
A^2^	0.001	1	0.001	0.2761	0.6155	
B^2^	0.0018	1	0.0018	0.483	0.5095	
C^2^	0.0356	1	0.0356	9.73	0.0169	

**Table 7:** Statistics analysis of the mechanical performance results.

Responses	Compressive Strength (MPa)	Compressive Rebound Modulus (MPa)	Flexural Strength (MPa)
Standard deviation	0.7885	84.13	0.0605
Mean	9.42	804.65	0.5024
*R* ^2^	0.9409	0.9601	0.9472
Adjusted *R*^2^	0.8649	0.9088	0.8794
Adequate precision	11.8993	16.1664	14.0826
Model type	Quadratic model	Quadratic model	Quadratic model
Model	Significant	Significant	Significant
Lack of fit	Insignificant	Insignificant	Insignificant
*F*-value	12.38	18.71	13.96
*p*-value	0.0016	0.0004	0.0011

**Table 8:** The gradation of LSCSM and CCSM.

Mesh Size (mm)	63	53	37.5	31.5	26.5	19	9.5	4.75	2.36	0.6	0.075
LSCSM Passing Rate (%)	100	100	70.94	48.82	44.34	44.34	44.08	19.46	12.66	9.01	3.28
CCSM Passing Rate (%)	-	-	-	100	95.4	87.9	65.9	38.2	24.5	13.6	2.7

## Data Availability

Data are contained within the article.
